# Insights into Cationic Transference Number Values and Solid Electrolyte Interphase Growth in Liquid/Solid Electrolytes for Potassium Metal Batteries

**DOI:** 10.1021/acsphyschemau.2c00024

**Published:** 2022-09-20

**Authors:** Jelena Popovic

**Affiliations:** Max Planck Institute for Solid State Research, Heisenbergstr. 1, 70569 Stuttgart, Germany

**Keywords:** battery, anode, potassium, solid electrolyte interphase, electrolyte

## Abstract

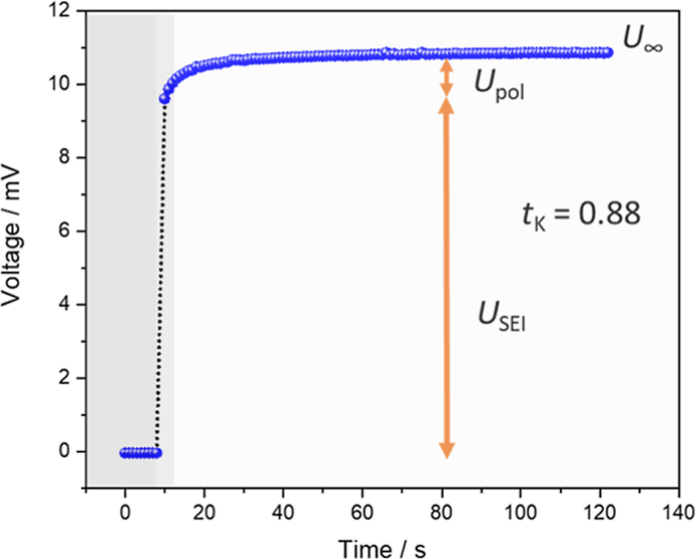

Liquid/solid battery electrolytes make separators dispensable and enable a high cationic transference number with liquid-like room temperature ionic conductivity. This work gives insights into electrochemical behavior (galvanostatic polarization and time-dependent impedance spectroscopy) of liquid/solid electrolytes containing potassium salts in battery cells enclosing potassium metal anodes. Very high potassium transference numbers (*t*_K_ = 0.88) are observed in carbonate-based electrolytes, linked with long-term mechanical instability of the solid electrolyte interphase on the potassium anode. In the case of glyme-based electrolytes, electrochemical behavior indicates the existence of the highly porous solid electrolyte interphase and additional surface porosity of the potassium electrode.

## 1 Introduction

Potassium batteries are currently considered a viable alternative option for substituting rechargeable lithium batteries due to a number of beneficial properties.^1−3^ However, although the specific theoretical capacity of K metal is substantial (687 mA h g^–1^), it is hardly attainable even in the lab-scale devices, due to the issues analogous to the Li and Na metal electrodes. These are related to the large volume expansion of the metal anode upon deposition, dendrite growth causing capacity loss and short circuits, and continuous solid electrolyte interphase (SEI) growth due to high reactivity of K.^4−6^ Recent experimental efforts have been focused on the preparation of carbon nanotube-based^7^ or potassium fluoride-rich^8,9^ artificial SEIs, and development of interlayers guiding uniform K plating such as polyvinyl alcohol–borax.^10^ The fundamental understanding of the differences in SEIs forming on different alkali metals is still necessary.^11^

Liquid/solid battery electrolytes are an interesting class of materials providing tunable cationic transference number linked with suitable room-temperature ionic conductivity (in the order of mS cm^–1^) and good wettability of nanostructured electrodes. Here, a monolithic porous and amorphous oxide (such as SiO_2_ or anodic aluminium oxide, AAO) acts both as a separator and as a surface-active material, enabling anion immobilization by specific adsorption and space charge formation.^12−14^ Adsorption may additionally be linked with the “overlimiting current” mechanisms in small (e.g., submicrometer-sized) pores, such as surface conduction and electro-osmotic hydrodynamic flow counteracting diffusion,^15,16^ or with current rectification in charged conical pores,^17,18^ leading to high-performance metal anode batteries.^19^

**Figure 1:**
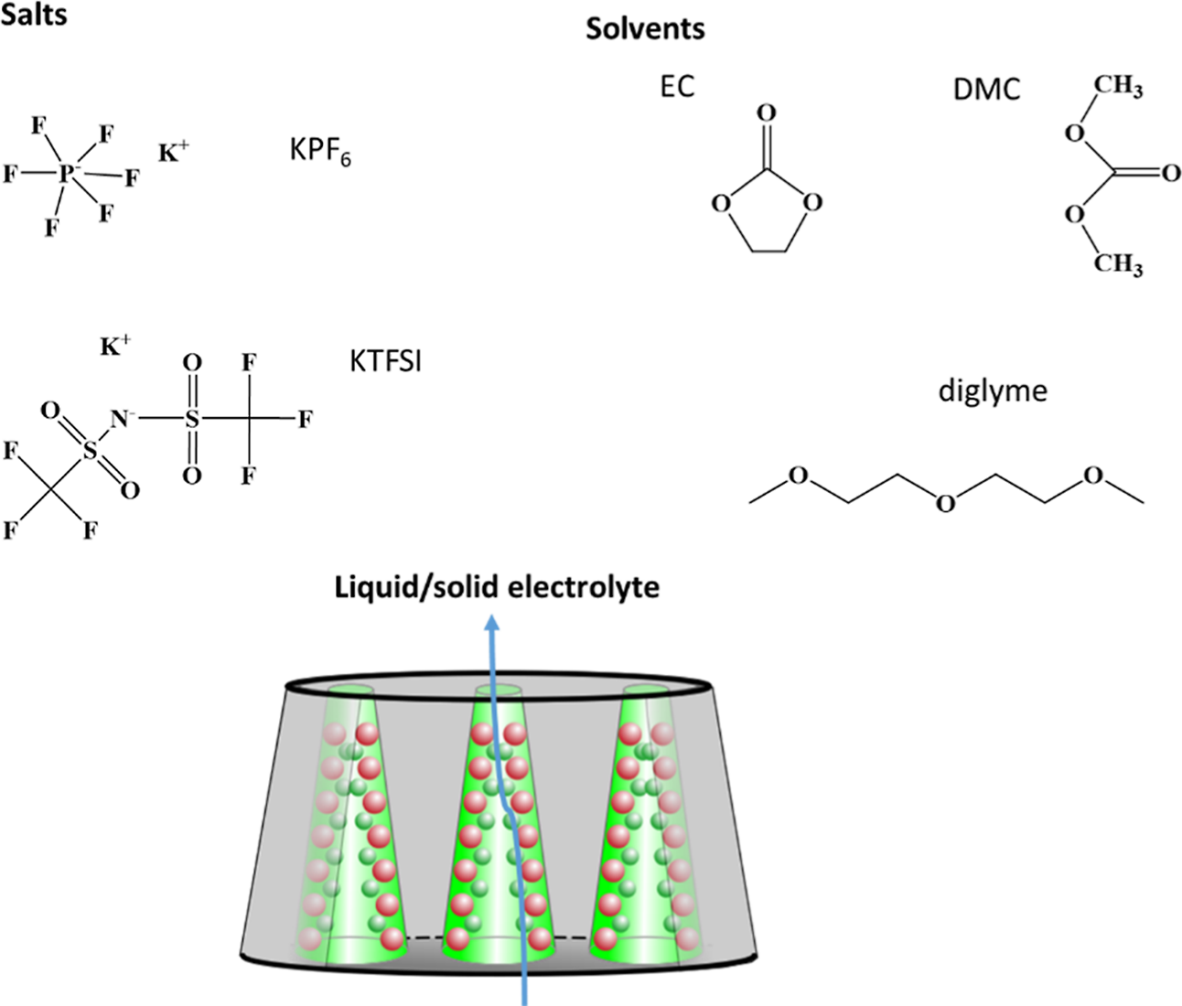
Materials and suggested K^+^-ion transport pathway. Chemical structures of salts (KPF_6_ and KTFSI) and solvents (ethylene carbonate, EC, dimethyl carbonate, DMC and diglyme) used in the study are shown in the upper part. The lower part shows the porous (e.g., conical pores) anodic aluminium oxide, AAO (gray) infiltrated with liquid salt-in-solvent electrolyte, where red spheres represent the adsorbed anions (PF_6_^–^ and TFSI^–^) and green spheres represent the mobile cation (K^+^). Blue pathways indicate the expected transport of K^+^ in the space charge zones formed on the pore surface.

In this paper, the focus lies on investigation of bulk (transference number) and interfacial (SEI growth under open circuit condition) electrochemical properties of two main classes (carbonate- and glyme-based) K–liquid electrolytes infiltrated in commercially available AAO with the abovementioned conical porosity (Figure 1, upper base radius of 20 nm and lower base radius of 200 nm^14^). These classes of liquid electrolytes are chosen as representative ones, since they are used in commercial battery cells and lab-scale alkali metal–sulfur or alkali metal oxygen cells. Two different salts of satisfactory solubility are chosen,^20^ as they are expected to guide the formation of unique SEI chemical composition. It has been previously shown by Wang et al. that potassium perchlorate (KPF_6_) leads to a more organic and potassium bis(trifluoromethanesulfonyl)imide (KTFSI) to more inorganic component-dominated SEI in both carbonate- and ether-based electrolytes.^21^ The results obtained from K symmetric cells are compared to similar cells containing Li and Na metals. SEI growth has been investigated under open circuit condition, which is of high relevance for cell storage and calendar ageing (e.g., corrosion).

## 2 Experimental Section

### 2.1 Materials Preparation

The preparation of materials and cells was performed in a glovebox (<0.1 ppm H_2_O, ≤0.1 ppm O_2_) with an Ar atmosphere. The liquid electrolytes were prepared by dissolving nominally 1 M of K-salt (KPF_6_, ≥99%; KTFSI, 97%) in the suitable solvents (EC, anhydrous, 99%; DMC, anhydrous, ≥99%; diglyme, anhydrous, 99.5%). The H_2_O content in the liquid electrolyte was controlled to be below 1 ppm using the Karl Fischer titration technique. Both liquid electrolytes were transparent solutions. For the preparation of liquid/solid electrolytes, liquid electrolytes were infiltrated into AAO (Whatman Anodisc, *d* = 13 mm) overnight. Materials characterization of AAO has been reported previously.^14^ A clean blade was used to cut fresh K electrodes from a K cube (cubes in mineral oil, 99.5% trace metals basis). All materials were purchased from Sigma-Aldrich.

### 2.2 Electrochemical Measurements

A custom-made copper-plated polytetrafluoroethylene cell with an adjustable screw was used for all electrochemical measurements. The electrochemical measurements were performed as soon as possible after the cell assembly (e.g., minutes after). Electrochemical impedance spectroscopy (EIS) was carried out in the potentiostatic mode in the frequency range from 10^–1^ to 10^7^ Hz using a Solartron 1260 frequency analyzer. The measurement amplitude was 10 mV, which is the lowest possible amplitude and thus closest to the open circuit potential condition. The data analysis was performed using a ZView software from Scribner Associates, version 3.5c. For the galvanostatic polarization, a Keithley 2604B source meter was used.

## 3 Results and Discussion

**Figure 2:**
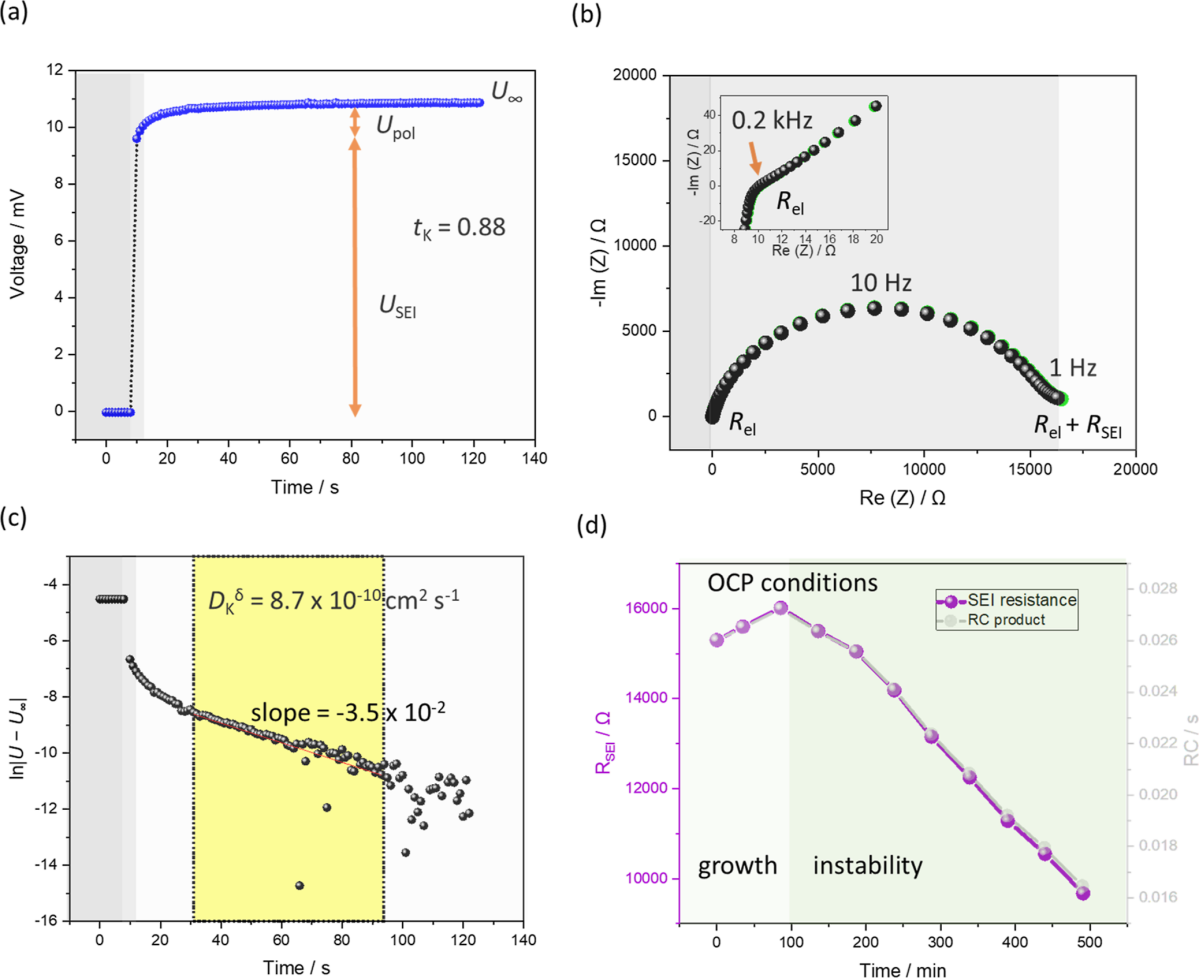
Potassium transference number (*t*_K_), potassium salt diffusion coefficient (*D*_K_^δ^), and SEI growth on K (*R*_SEI_) determined from K/AAO:(KPF_6_/EC + DMC)/K symmetric cells. (a) Galvanostatic polarization experiment (*I* = 6 × 10^–7^ A, blue points) showing the contribution of the SEI (*U*_SEI_) and concentration polarization of the electrolyte (*U*_pol_) to the total voltage response. (b) Nyquist plot corresponding to the EIS measurement performed before (black) and after (green) the galvanostatic polarization experiment. The visible semicircle corresponds to the non-changing SEI resistance (*R*_SEI_), while the first *x*-axis intercept corresponds to the contribution of the bulk electrolyte resistance (*R*_el_). (c) Salt diffusion coefficient determination from the long polarization time (25–90 s, marked in yellow). (d) Time-dependent change in *R*_SEI_ measured under open circuit potential. The gray and green scales indicate different time regimes of polarization and SEI growth.

**Figure 3:**
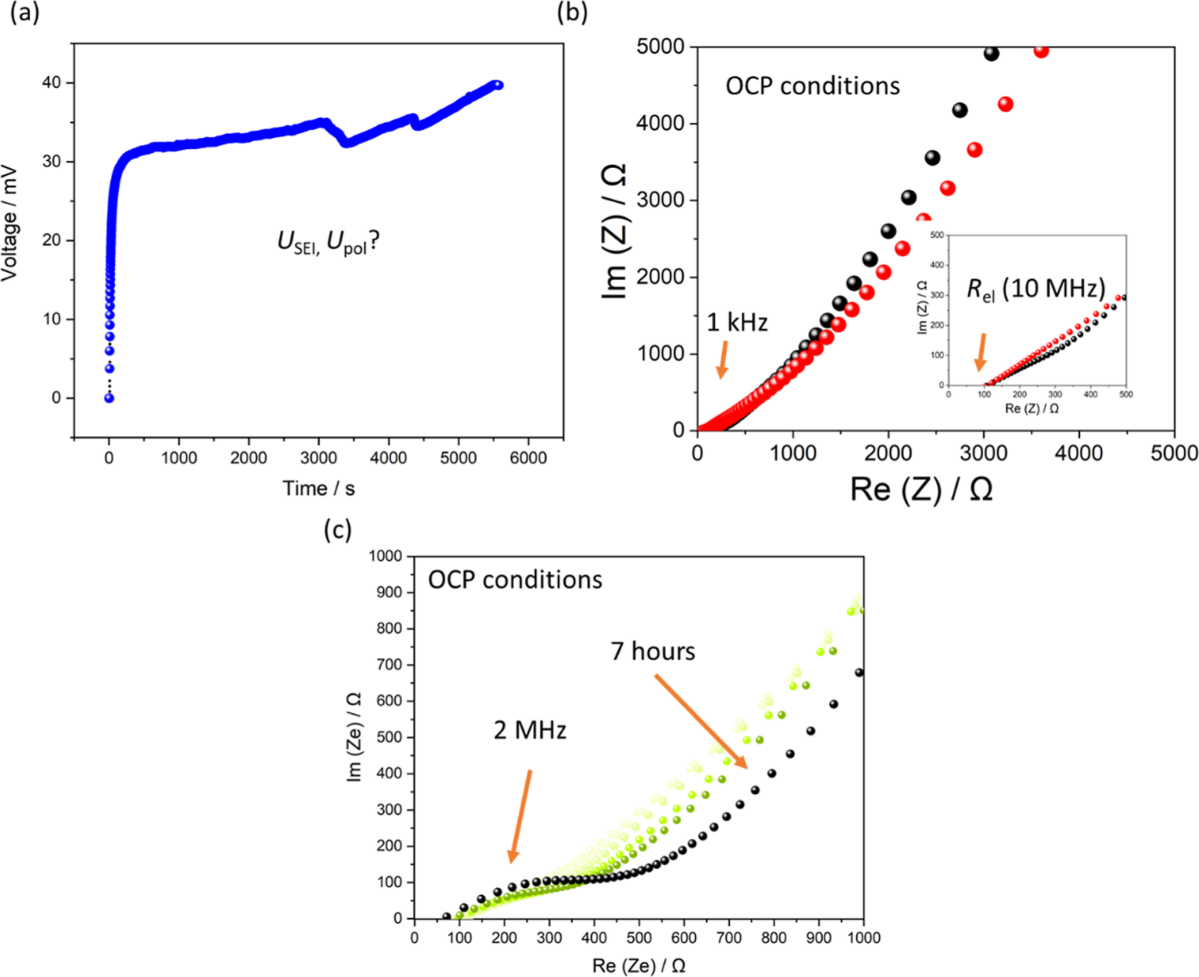
Galvanostatic polarization and EIS data measured on K/AAO:(KTFSI/diglyme)/K symmetric cells. (a) Galvanostatic experiment with 1 nA current shows that steady-state has not been achieved after 5000 s. (b) Contribution of *R*_SEI_ is not visible before (black dots) and after (red dots) the galvanostatic polarization of cells. (c) Observed EIS features change after 7 h of aging under open circuit conditions. These most probably still originate from a transport through the highly porous electrode as described by a porous electrode mode.

The investigated symmetric cells (e.g., both working and counter electrodes are made of the K metal) always showed an open circuit potential in the proximity of zero value (Figures 2 and 3), as expected for symmetric cells and unlike previously reported by Hosaka et al.^22^ The Hebb–Wagner method of stationary polarization has been widely used for determining the conductivity of electrons and holes in solid-state electrolytes.^23^ Recently, it was shown that a similar concept can be employed for determining the cationic transference number of soft matter electrolytes, when anion-blocking and cation-containing electrodes are used.^13^ Unlike the potentiostatic method first reported by Evans et al., the galvanostatic method circumvents the issue of determination of the initial current response.^24^

In the room-temperature galvanostatic polarization experiment with AAO:KPF_6_/EC + DMC electrolyte sandwiched between two K electrodes, the salt concentration gradient forms very quickly (already after 25 s, Figure 2a). Since the SEI contribution remains constant during such short time (*U*_SEI_ = *IR*_SEI_, corresponding to the semicircle visible in the Nyquist plot, Figure 2b), the polarization response (e.g., cationic transference number) was determined from the ratio of the initial (*U*_SEI_) and the steady-state voltage value (*U*_∞_). The contribution of the electrolyte resistance to voltage jump shown in Figure 2a is too small to be visible, *IR*_el_ = 6 × 10^–6^ V. The calculated value of potassium transference number, *t*_K_ = 0.88, suggests predominant cationic conduction and is much higher than the transference number of the liquid electrolyte (≤0.5^25,26^) and the lithium counterpart (*t*_Li,pol_ = 0.6 to 0.4 in the 0.5 to 1 M LiCF_3_SO_3_ in triglyme^14^). It is to be noted that refs (25) and (26) report the potassium transference number from Hittorf and moving boundary type of experiments on low concentrations of KBr and KCl in water. At higher salt concentrations, the value of cationic transference number is expected to drop slightly, as shown by investigation of the thermodynamic parameters.^27^ The comparison between the lithium and potassium electrolytes is fair, since the adsorption effect is expected to be much lower in the carbonate-based electrolytes than glymes, due to their higher dielectric constant (ε = 25 for EC/DMC vs ε = 8 for triglyme^28,29^).^12^ Even though a high potassium transference number indicates a considerable improvement in the strength of the anion adsorption or ion rectification, no increase of the effective bulk ionic conductivity compared to the liquid electrolyte can be reported.

Using a parallel switching ionic conduction model for the situation when no considerable increase of the ionic conductivity in the liquid/solid electrolyte to the bulk is seen, the effective transference number may be expressed as *t*_K,eff_ = 1 – β_∞_(1 – φ)(1 –*t*_∞,+_), where β_∞_ corresponds to the proportion of bulk liquid pathways contributing to the overall conductivity, φ to the volume fraction of the solid (in this case 0.24^14^), and *t*_∞,+_ to the cationic transference number of the liquid electrolyte.^12^ If *t*_∞,+_ = 0.5 is assumed as reported in ref (25) and *t*_K_ ≈ *t*_K,eff_ (e.g., there are no other considerable effects on ion transport than space charge zone formation at the liquid/solid interface), β_∞_ = 0.3 is calculated. Such values of β_∞_ show that, even under conditions of strong adsorption, structural issues such as non-continuous pores of AAO are of importance. The non-continuous pores are thus not only detrimental for the ion transport on the walls but also prove to be an important obstacle for the transport pathways in the bulk of liquid electrolyte.^12^ For reaching the β_∞_ = 1 value, improvement of morphology is necessary—pores should preferably be continuous and straight.

The salt diffusion coefficient was determined from the long polarization time (25 to 90 s) from , where *L* is the electrolyte thickness (here 15 μm) and τ^δ^ is the salt polarization constant related to the inverse of the absolute value of the slope marked in yellow in Figure 2c (ln|*U* – *U*_∞_|/mV–time dependence). The obtained value, *D*_K_^δ^ = 8.7 × 10^–10^ cm^2^ s^–1^, is 2 orders of magnitude lower than the ones in lithium-based liquid/solid electrolytes and 1 order of magnitude lower than isothermal diffusion coefficients in KCl–H_2_O electrolytes at comparable salt concentrations.^30^ The observed value indeed indicates a possible existence of higher order ion pairs and aggregates that contribute to the potassium transport, as seen from the high value of *t*_K_, but exhibit a decreased mobility compared to the solvated free ions, the mobility of which is expected to be enhanced compared to the Li case.^31^ Future studies should involve surface-sensitive infrared spectroscopy for elucidation of the molecular structure of the solid/liquid electrolytes.

The crossover current can be estimated from the theoretical consideration of the space charge zone formation at the K electrodes using , where *e* is the electron charge, *c*_0_ is the initial salt concentration, *D*_app_ is the apparent chemical diffusion coefficient of the salt in solvent, *t*_–_ is the anion transference number, and *L* is the distance between the two electrodes.^32,33^ For the *c*_0_ = 6 × 10^20^ cm^–3^, J* = 0.2 mA cm^–2^ is obtained, a value slightly lower than experimentally found crossover currents for the liquid KPF_6_–carbonate electrolytes.^4^

As already stated, the *R*_SEI_ value remained unchanged during the galvanostatic polarization measurement. The maximum frequency associated with this semicircle (*f*_max_ = 10 Hz) is 10 times higher than the one observed for Li and Na in contact with liquid carbonate-based electrolytes,^34^ while the values of capacitances ( = 9.9 μF) are comparable to the ones found for Na in contact with carbonate-based electrolytes. Thus, considerable SEI porosity cannot be excluded in this case, further complicating the evaluation of the impedance data beyond the simple RC equivalent circuit.^34,35^ After the initial phase of apparently linear SEI growth suggesting surface-controlled mechanism,^36,37^ a long period of decrease of *R*_SEI_ is observed. This may be due to the (i) formation of more conductive SEI phases or their interfaces in time^38^ and (ii) mechanical instabilities of the SEI due to its detachment in the course of continuous chemical reaction. The solubility of the SEI should not be a considerable issue in K-based SEIs, at least not for the formed inorganic SEI constituents, as shown by Moshkovich et al.^39^ Since the *R*_SEI_ change is comparably large (ca. 20% in the course of 500 min, Figure 2d) and the number of K compounds typically forming in the SEI on K is even higher than that in the Li or Na case,^6^ the second option (e.g. mechanical instability of the SEI) seems more probable. The evolution of the relaxation times, τ = *R*_SEI_*C*_m_, follows the time-dependent change of the *R*_SEI_, since *C*_m_ values do not change significantly (Figure 2d).

It proved to be difficult to determine *t*_K_, values for AAO:(KTFSI/diglyme) using the galvanostatic polarization method in the symmetric K cell. First, the potential value seems not to reach the steady state, even after 5000 s (Figure 3a) of constant current application. The logical explanation for this behavior would be a strong evolution of the SEI. Indeed, in Figure 2a, the SEI contribution to the potential jump is the dominant one. However, here the impedance contribution of the SEI seems not even to be present in the Nyquist plot before and after the polarization (Figure 3b) as only one *x*-axis intercept (Figure 3b, inset) is visible and no clear semicircle, which is another important difference to the cells with carbonate electrolytes discussed previously. The *x*-axis intercept is most probably corresponding to *R*_el_, as the resultant frequency is comparably high (*f*_intercept_ = 10 MHz). The apparent non-existence of the SEI in ether-based electrolytes has already been speculated for graphite anodes in contact with the K-based electrolyte.^40^ Nevertheless, compared to the graphite, where SEI formation is expected only at voltages around zero, K is considerably more reactive and forms SEIs even under open circuit conditions. Another, more valid option, would be the existence of highly porous SEI, for which the impedance response would be rather Warburg-like, if the *R*_SEI_ value is negligible and collections of electrode pores are considered, according to the porous electrode model (Figure 3).^41^ Such highly porous SEIs have been observed in glyme-based electrolytes in contact with Na electrodes directly upon cell assembly.^34^

The apparent change in the phase angle in the impedance data before (black dots) and after (red dots) galvanostatic polarization (Figure 3b, inset) indicate a change in the smoothness of the K electrode.^42^ It appears that solvent, rather than the salt, plays a crucial role in formation of porous SEIs, as here KTFSI is employed rather than the sodium triflate. Indeed, the linear glymes are known to more easily form SEI compounds by polymerization/oligomerization,^43^ which may induce porosity.

As pointed out in the porous electrode model, the galvanostatic polarization experiment in such electrodes would lead to a steady-state situation only at very long times (*t* → ∞, yet not reached in Figure 2a) as the potential in the pore would change very slowly, regardless of the pore size distribution.^44^ This is an important distinction between porous SEIs on K and Na, as galvanostatic polarization was possible in the latter case. The features observed in the Nyquist representation slowly change in the course of 7 h of cell aging under open circuit conditions (Figure 3c). The newly appearing semicircle also fits well to the theory of porous electrodes. However, additional contribution of concentration polarization in the SEI pores should also not be fully excluded, as the necessary estimation of the capacitance for the observed semicircle is not possible here.

## 4 Conclusions

In summary, the galvanostatic polarization and EIS response of two different K liquid/solid electrolytes, namely KPF_6_/EC + DMC and KTFSI/diglyme in AAO, have been investigated using symmetric K cells. The behavior of these two electrolytes is strikingly different. An extraordinarily high potassium transference number has been found for (KPF_6_/EC + DMC):AAO (0.88), indicating that the mechanism of potassium ion transport is similar to the ones already observed for comparable lithium electrolytes. However, in the potassium case, the anion adsorption on AAO pore walls appears to be much stronger, even at a salt concentration close to the solubility maximum. With the (KPF_6_/EC + DMC):AAO electrolyte, the formed SEI on K seems to be unstable after longer wait times at open circuit potential, as reflected by the considerable SEI resistance drop after 8 h of cell storage.

The galvanostatic polarization and EIS data on (KTFSI/diglyme):AAO symmetric cells suggest that both the formed SEI and the surface of K electrode are highly porous. Such nanoporous SEIs have already been reported for Na in contact with glyme-based electrolytes with a different salt, suggesting that the solvent plays a crucial role in the determination of the final SEI morphology. The resistance of such a liquid/solid SEI remains negligible even after 7 h of open circuit aging. Galvanostatic polarization of cells with (KTFSI/diglyme):AAO did not lead to the steady state even after 1.5 h. This is in line with the theory of porous electrodes, stating that such behavior is a reflection of very slow concentration polarization in the pores. Further studies should involve morphological investigations of the K electrode surface after ageing (e.g., atomic force microscopy) and of the SEI nanoporosity (e.g., cryo-transmission electron microscopy). Both techniques are challenging, due to the air sensitivity of both K-metal and its corresponding SEI.
